# Highlighting computations in bioscience and bioinformatics: review of the Symposium of Computations in Bioinformatics and Bioscience (SCBB07)

**DOI:** 10.1186/1471-2105-9-S6-S1

**Published:** 2008-05-28

**Authors:** Guoqing Lu, Jun Ni

**Affiliations:** 1 Department of Biology, University of Nebraska, Omaha, NE 68182, USA; 2 Department of Radiology, University of Iowa, Iowa City, IA 522542, USA

## Abstract

The Second Symposium on Computations in Bioinformatics and Bioscience (SCBB07) was held in Iowa City, Iowa, USA, on August 13–15, 2007. This annual event attracted dozens of bioinformatics professionals and students, who are interested in solving emerging computational problems in bioscience, from China, Japan, Taiwan and the United States. The Scientific Committee of the symposium selected 18 peer-reviewed papers for publication in this supplemental issue of *BMC Bioinformatics*. These papers cover a broad spectrum of topics in computational biology and bioinformatics, including DNA, protein and genome sequence analysis, gene expression and microarray analysis, computational proteomics and protein structure classification, systems biology and machine learning.

## Introduction

Bioinformatics is an emerging interdisciplinary field that evolves very rapidly, with new research content, such as translational bioinformatics, constantly being added to the already long list of topics. The mission of the Symposium of Computations in Bioinformatics and Bioscience (SCBB) is to provide a regular forum for researchers who are interested in computations in bioscience and bioinformatics to share their research experience and achievements, discuss various issues pertaining to biological computation and software development, and strengthen existing and foster future research collaborations. The first symposium (SCBB 2006) was held in Hangzhou, China and 26 peer-reviewed papers were selected for publication in the *BMC Bioinformatics *special issue [1].

The Second Symposium on Computational Biology and Bioinformatics (SCBB07) in conjunction with the International Multi-Symposiums on Computer and Computational Sciences 2007 (IMSCCS|07) was held at the University of Iowa, Iowa City, Iowa, USA, on August 13–15, 2007. Besides regular scientific presentations, the symposium had four keynote presentations addressing pressing issues, from high performance computation to genome annotation. The symposium also hosted a tutorial section on nanotechnology and image processing. This special issue consists of 18 peer-reviewed papers authored by research scientists, faculty and graduate students in different disciplines, with diverse backgrounds, from 30 institutions in four different countries or regions.

## Process of submission and reviews

All manuscripts were electronically submitted through EasyChair, a conference management system (). Each manuscript was critically reviewed by at least two referees. The quality of each paper was evaluated on the significance of its contribution to computational biology and bioinformatics, its technical novelty and its rigor in methodology. The selected papers published in this special issue cover a broad range of topics and can be divided into the following major categories:

## DNA, protein and genome analyses

Genome comparison has become an essential means of determining the function of genes and non-coding regions of the genome. Yang et al. report their findings on bidirectional promoters, i.e., the regulatory regions shared between two consecutive genes, in eight vertebrate model organisms [2]. Their mapping study shows that many bidirectional promoters occurred after the divergence of chicken and fish. This observation is important because it leads to a sound understanding of bidirectional promoter evolution.

Identifying DNA-binding proteins is an important step towards unveiling the complexity of gene regulatory networks. Chang et al. introduce a promising approach that employs the profiling of evolutionarily conserved residues revealed in DNA-protein contact domains to predict whether a domain or a protein can be bound to DNA [3]. Their approach was found to be highly sensitive, with the capability of identifying all positive samples, but did not lose its high selectivity, with a false positive rate of less than 5%.

Sequence comparison as a fundamental bioinformatics operation has many applications such as gene finding and protein function annotation. Lu et al. proposed an improved string composition method to quantify evolutionary information in genetic sequences for further sequence comparison [4]. Using simulated and experimental datasets, they show that their proposed method is more robust than existing counterparts and comparable in robustness to alignment-based methods.

Inference of phylogeny is one common task faced by many biologists. There is no optimal solution, however, in the construction of phylogenetic trees. Guo et al. propose a new method called p-ECRNJ that uses a neighbor joining (NJ) algorithm to refine unresolved nodes generated from p-Edge Contraction and Refinement (p-ECR), a topological transformation approach [5]. It is concluded by the authors that this enhanced method can efficiently improve local topological transforms and result in better evolutionary trees.

## Micorarray and gene expression analyses

Microarray analysis as a high-throughput technology has brought us not only many unprecedented opportunities, such as monitoring expression profiles of tens of thousands of genes simultaneously, but also many unseen challenges, such as data processing and analysis, and molecular pathway discovering. How to find biologically significant genes and discover co-regulated gene networks using the methods and exploratory techniques of inferential statistics is still the most compelling topic in this year's symposium.

Deng et al. propose an Intersection-Union Tests (IUT) adjustment procedure, called Relaxed IUT, to identify statistically significant genes in treatment versus control samples [6]. Simulation and real case studies highlight the advantages of this new procedure over traditional IUT. It is less conservative and more powerful for intersecting independent tests than the traditional Venn diagram approach. Zeng et al. introduce a dimension reduction methodology that combines feature extraction with redundant gene elimination for tumor classification [7]. A novel metric of redundancy was proposed by the authors to eliminate redundant genes before feature extraction using the discriminative ability of each gene and pairwise complementarities. The analytical result of two microarray datasets illustrates that this innovative method is more effective and reliable in dimension reduction.

Clustering is a powerful exploratory technique for the discovery of co-regulated genes and gene networks in microarray data analysis. Wu introduces a genetic weighted k-means algorithm that combines a genetic algorithm and a weighted k-means algorithm for large-scale gene expression data clustering analysis [8]. The analytical result of both synthetic and real gene expression datasets shows that this improved algorithm is superior to the traditional k-means algorithm. There are many clustering algorithms for microarray data analyses; however, it remains an open question which clustering algorithm is more reliable. Wilkin and Huang compare the Lloyd's k-means clustering method with the progressive greedy k-means clustering method, using both randomly-generated and experimental datasets, for running time and distance efficiency [9]. Their study concludes that the Lloyd's k-means clustering algorithm is more efficient; however, the conclusion may change in different circumstances.

There is growing interest in identifying gene pathways, i.e., a group of genes interacting with each other to perform certain biological functions, in the study of diseases. Liang et al. introduce a method based on the concept of fuzzy set theory to measure the significance of gene pathways in disease [10]. Their experiment, with published diabetic gene expression datasets, together with a list of predefined pathways, shows that their approach provides a feasible solution to the general problem of measuring the difference between two groups of datasets.

The contents and data models in microarray repositories are largely heterogeneous, which makes it difficult to perform a database search. Stokes et al. introduce ArrayWiki, a computer program that resolves the above-mentioned problem by uniting disparate meta-data regarding microarray meta-experiments [11]. The software contains many attractive features including a friendly knowledge management interface and a programmable interface, which can be accessed at .

## High performance computing

High-performance computing (HPC) describes integrated computer systems developed for and capable of performing large amounts of computation quickly. Chin et al. introduce a computational framework named the Biological Graph Environment (BioGraphE) that connects graph problems in biology to computational solvers and high-performance systems for graph analysis [12]. It can automatically identify and deploy complex graph algorithms and integrate those algorithms with powerful and efficient computational solvers and HPC systems. This platform brings high-performance software and hardware capabilities to solve challenging graph problems without requiring biologists to know much about specific computing environments.

Govil et al. describe MLIP, a multiprocessor two-point genetic linkage analysis system that supports statistical calculations based on the full parameter space implicit in the linkage likelihood [13]. Both simulated and real experimental datasets are analyzed using MLIP and the results show it significantly speeds up linkage calculations over a grid space of model parameters. With MLIP, full multidimensional genome scans can be accomplished within a reasonable timeframe. Mishima et al. employs a queuing scheduler built on Grid Engine and runs on a Rocks Linux cluster for the analysis of statistical genetics [14]. Their experiment with a large dataset of loci and families shows that the deployment of exhaustive haplotype analyses using non-parallel software on a Linux-based system is an effective and efficient approach in terms of both cost and performance.

## Computational proteomics

Tandem mass spectrometry has emerged as a fundamental, high throughput proteomic technique, owing in part to the successful application of database searching and de novo sequencing algorithms. Wu et al. describe a novel method that can be used for assessing the quality of tandem mass spectra for better annotation [15]. In their computational experiment with tandem mass spectra datasets acquired by ion trap mass spectrometers, this new method is able to eliminate the majority of poor quality spectra with a slight loss of high quality spectra. This shows the outperformance of the proposed method compared to existing ones. Their method is applicable to assessing the quality of spectra acquired by virtually any instrument, not just limited to ion trap mass spectrometers.

## Protein structure classification

Protein function is mainly determined by its structure. Gu et al. introduce a protein structural classification method that integrates information and probability theories together with a long-term correlation consideration [16]. A residue occurrence frequency is used instead of physiochemical indices for calculating long-term correlations, whereas the statistical strategy of residual occurrence frequency is changed from a single sequence to a whole-training dataset. Both re-substitution and cross-validation tests show that this new method significantly improves the accuracy of protein structure classification.

## Systems biology

Systems biology is a relatively new field that focuses on the systematic study of complex interactions in biological systems. Arikuma et al. propose a new Ontology-Driven Hypothetic Assertion (OHA) framework that includes pathway generation, drug interaction detection, simulation model generation, numerical simulation, and hypothetic assertion [17]. Their study results demonstrate that the OHA framework is a promising approach for *in silico* prediction of drug interactions. Many numerical solvers currently used in systems biology are often ill-conditioned due to stiffness. Quo and Wang [18] describe new optimal numerical solvers by systematically comparing qualitative and quantitative performance metrics. The classic Belousov-Zhabotinskii (BZ) reaction described in the Oregonator model is studied and two general rules are found for selecting optimal numerical solvers for stiff, complex oscillatory systems. Their experiment provides insights into the systematic study of a variety of molecular-level models of biomedical systems for human disease diagnosis, which will lead to a better understanding and prediction of disease mechanisms and progression in therapeutic treatments.

## Machine learning

The quantitative structure activity relationship (QSAR) modeling of drug molecules enables one to predict molecular activities and thus reduces the cost of traditional experiments needed for drug design. Li et al. introduce a strategy called asymmetric bagging to deal with the issue of unbalanced samples where the number of drug molecules is relatively small [19]. A new algorithm is developed to remove redundant and irrelevant features of drug molecules for the analysis of asymmetric bagging. Their computational results with molecular activity data show that the asymmetric bagging strategy improves sensitivity values regarding molecular activities and the feature selection algorithm enhances prediction ability pertaining to molecular activities.

## Future meeting

The Symposium on Computations in Bioinformatics and Bioscience is an annual conference. The third symposium is scheduled to be held in Shanghai Jiaotong University, Shanghai, China on 18-20 August 2008. The updated information about the next symposium can be found at the Web site: .

## Competing interests

The authors declare that they have no competing interests.
